# The Reliability and Validity of Short Online Questionnaires to Measure Fruit and Vegetable Intake in Adults: The Fruit Test and Vegetable Test

**DOI:** 10.1371/journal.pone.0159834

**Published:** 2016-07-21

**Authors:** Jolien Plaete, Ilse De Bourdeaudhuij, Geert Crombez, Saidja Steenhuyzen, Liesbet Dejaegere, Erika Vanhauwaert, Maïté Verloigne

**Affiliations:** 1 Department of Movement and Sports Sciences, Ghent University, Ghent, Belgium; 2 Department of Experimental-Clinical and Health Psychology, Ghent University, Ghent, Belgium; 3 Flemish Institute for Health Promotion and Disease Prevention (VIGeZ), Brussels, Belgium; 4 Knowledge and Innovation Center FOOD, University Colleges Leuven-Limburg, Leuven, Belgium; University of Oxford, UNITED KINGDOM

## Abstract

The first aim of this study was to investigate the stability of the Fruit Test and Vegetable Test over time and whether the Fruit Test and Vegetable Test are capable of measuring fruit and vegetable intake with consistency. Second, the study aimed to examine criterion (concurrent) validity of the Fruit Test and Vegetable Test by testing their agreement with 7-day food diary-derived measures of fruit and vegetable intake. In total 58 adults (31% male, mean age = 30.0±12.09y) completed the Flemish Fruit and Vegetable test by indicating the frequency of days that they ate fruit and vegetables and the number of portions during the past week. Validity was tested by using a 7-day food diary as a golden standard. Adults were asked to register their fruit and vegetable intake daily in a diary during one week. Spearman correlations were measured to compare total intake reported in the Fruit and Vegetable Test and in the 7-day diary. Agreement plots were used to illustrate absolute agreement. Test-retest reliability was evaluated by having participants completing the Fruit Test and Vegetable Test twice. The Fruit Test (ICC = 0.81) and Vegetable Test (ICC = 0.78) showed excellent and substantial reliability. The Fruit Test (ρ = 0.73) and Vegetable Test showed good validity. Agreement plots showed modest variability in differences between vegetable and fruit intake as measured by the Vegetable and Fruit Test and the 7-day food diary. Also a small underestimation of fruit intake in the Fruit test and vegetable intake in the Vegetable test against the 7-day food diary was shown. Based on the results, it is suggested to include portion size pictures and consumption of mixed vegetables to prevent underestimation. To prevent overestimation, it is concluded to add a moderate number of representative fruit and vegetable items, questions on portion size, household sizes with sufficient detail and food items highly tailored to the dietary behaviors and local food items of the population surveyed. The questionnaires can easily be adapted for the use in other diets (e.g. Asian diet), but reliability and validity should then be examined again. Also, validity remains to be tested in other population groups (i.e. low socio economic status groups, other age groups).

## Introduction

The World Health Organisation (WHO) recommends adults to consume a minimum of 400 g of fruits and vegetables per day to prevent chronic diseases (e.g. diabetes) [1]. However, in most Western countries a large part of the adult population does not meet this recommendation. In 2013, only 30% of Belgian adults ate two pieces of fruit per day (i.e. 250 gram), and only 39% ate two portions (i.e. 300 gram) of vegetables every day[2]. To address these public health concerns, effective interventions promoting fruit and vegetable intake (FV intake)in a large population are necessary. Computer-tailored interventions have shown to be effective in promoting dietary intake and are able to reach large populations at a low cost[3]. In computer-tailored interventions, online self-administered questionnaires are used to provide tailored feedback[4]. To ensure feasibility of computer-tailored interventions, attractive and brief online questionnaires are required[5–10]. These online questionnaires, however, also need to measure FV intake in a valid and reliable manner to allow adequate feedback based upon a comparison of adults’ mean intake of fruit and vegetables with the health guidelines (i.e. consuming a minimum of 400 g of fruits and vegetables per day) [5]. Kim and Holowaty (2003) conducted a literature review of brief, validated survey instruments measuring self-reported fruit and vegetable consumption. They identified survey instrument characteristics that are associated with greater validity and/or reliability[10]. These instruments were validated by comparing them to other methods (e.g. extended food frequency questionnaires, weighted dietary records, single or multiple 24-hour recalls). The authors found 10 instruments with less than 17 items, with correlations ranging from r = 0,29 to 0,84[10]. Through a further search using the International Register of Validated Short Dietary Assessment Instruments[11], 16 other short (<20 items) questionnaires that were not included in the review of Kim and Holowaty (2003) were identified. A variation in correlations coefficients was found, showing moderate [8, 12–14] to good validity [5, 8, 15–18] of the questionnaires. However, none of these instruments or the instruments reviewed by Kim and Holowaty (2003) were computerised or online questionnaires. Internet use is strongly increasing and new technologies for dietary assessment offer important advantages (e.g. easy processing of data, producing immediately results and increasing privacy and confidentiality)[19]. Therefore, it is necessary to also develop valid and reliable, brief online questionnaires that measure FV intake. The Fruit Test (FT) and Vegetable Test (VT) evaluated in this study, are brief, online questionnaires developed by the Flemish institute for Health Promotion and Disease Prevention (VIGeZ) to measure vegetable intake and fruit intake in Flemish adults[20]. A pre-test of the FT and VT was conducted to ensure clarity and ease of completion/interpretation of the FT and VT[20]. Furthermore, as suggested by Kim and Holowaty (2003), a moderate number of representative fruit and vegetable items, questions on portion size, food items highly tailored to the dietary behaviors and local food items of the population surveyed were included in the VT and FT[10]. In Belgium, these questionnaires include different kinds of fruit and vegetables frequently eaten in a Western diet, based on Belgian food consumption data [21–23]. Which makes the questionnaire highly tailored to local dietary behaviors and food items. Still, the measures are short (<16 items) and assess FV intake over seven days (the past week). Both the FT and VT were already evaluated by 116 and 70 adults, respectively. Adults positively scored the FT and VT with regard to feasibility and acceptability (good length, time needed, good structure, clear and relevant questions, easy to fill in and easy to understand)[20]. Despite these promising results, the reliability and validity of the FT and VT needs further scrutiny, to justify their use in computer-tailored programs. The first objective of this study was to investigate the stability and consistency of the FT and VT over time. Therefore, the test-retest reliability of the FT and the VT in Flanders was examined[24]. The second objective of the present study was to examine concurrent criterion validity of the FT and VT in Flemish adults by testing their agreement with 7-day food diary-derived measures of FV intake.

If the questionnaires would be sufficiently reliable and valid to measure FV intake, they can be considered useful for future research and interventions. Since suggestions to enhance reliability and validity were followed[10], we hypothesised the FT and VT to be valid and reliable online questionnaires.

## Methods

### Participants and procedure

Convenience sampling was used to recruit adults (> = 18 years) by handing out information letters to friends, family and university students. Participants who agreed to participate, signed an informed consent and received an email with more information on the study. The study lasted for three weeks and was conducted in September 2014. In week one, adults completed the FT and VT for the first time. In the second week, adults completed the FT and VT for the second time. Each time, adults first completed the FT and then immediately the VT. In week three, adults recorded their FV intake in a 7-day food diary. Email reminders were used to remind participants to fill in the 7-day food diary, the FT and VT. *Test-re-test reliability* was evaluated by having participants fill in the FT and VT twice with a time-interval of one week. Based on previous studies, the questionnaires were *validated* against a previously validated 7-day food diary [25, 26]. The study was approved by the Ghent University Ethics Committee (approval number: B670201422475).

### The Fruit test and the Vegetable test

The FT and the VT were developed by the Flemish institute for Health Promotion and disease prevention (VIGeZ) in Belgium. This study was conducted in the context of the eHealth intervention ‘MyPlan 1.0’, a computer-tailored intervention based on self-regulation that aims to increase FV intake of Flemish adults. We aim to incorporate the FT and VT in ‘MyPlan 1.0’ to measure mean FV intake per week and to provide feedback in which the mean FV intake of adults’ is compared with health guidelines[27].

**Fig 1:**
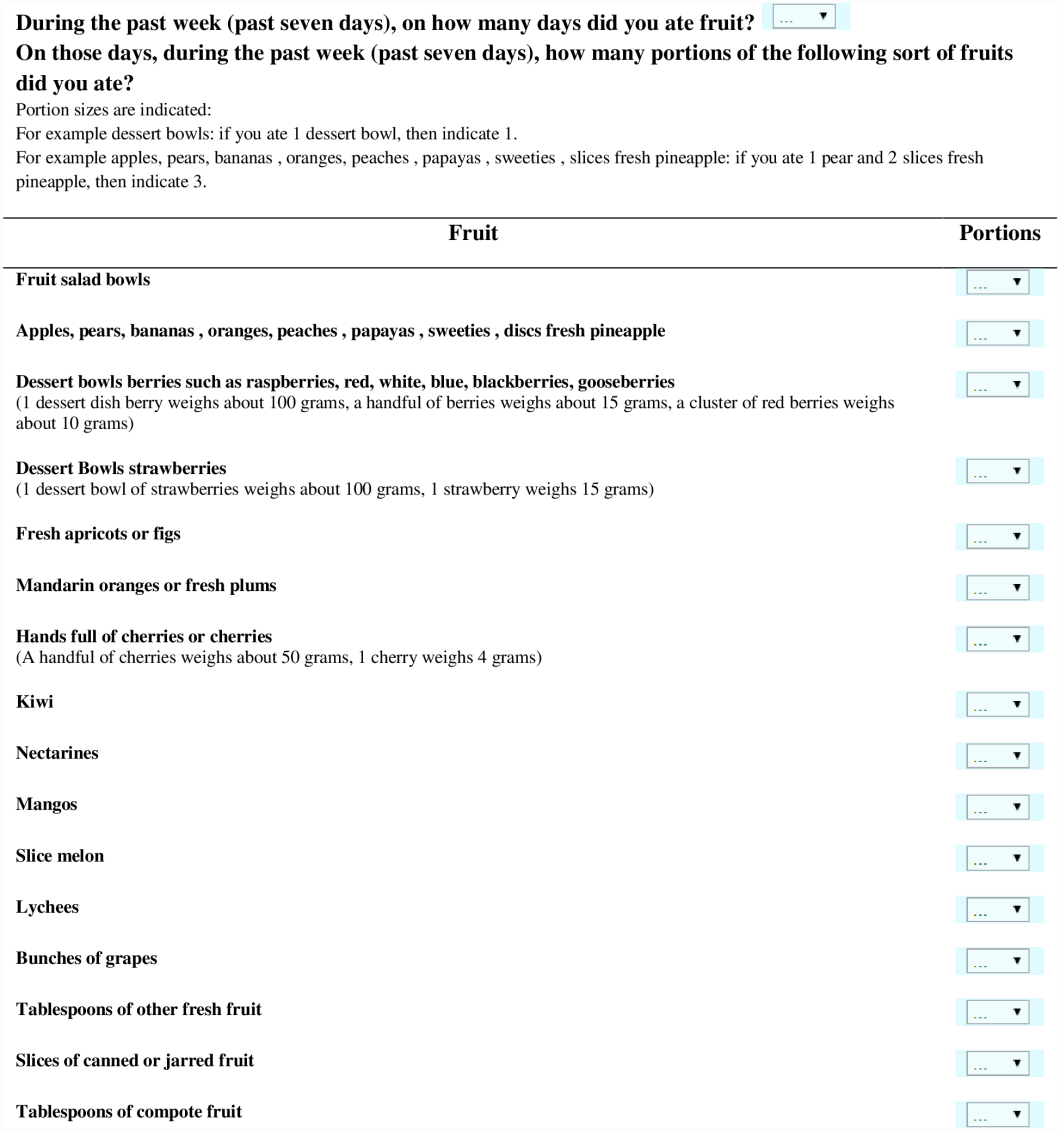
Fruit test. Items in the Fruit Test are grouped based on similar portion sizes. Via the dropdown, participants can indicate the amount of portions of fruit they ate.

**Fig 2:**
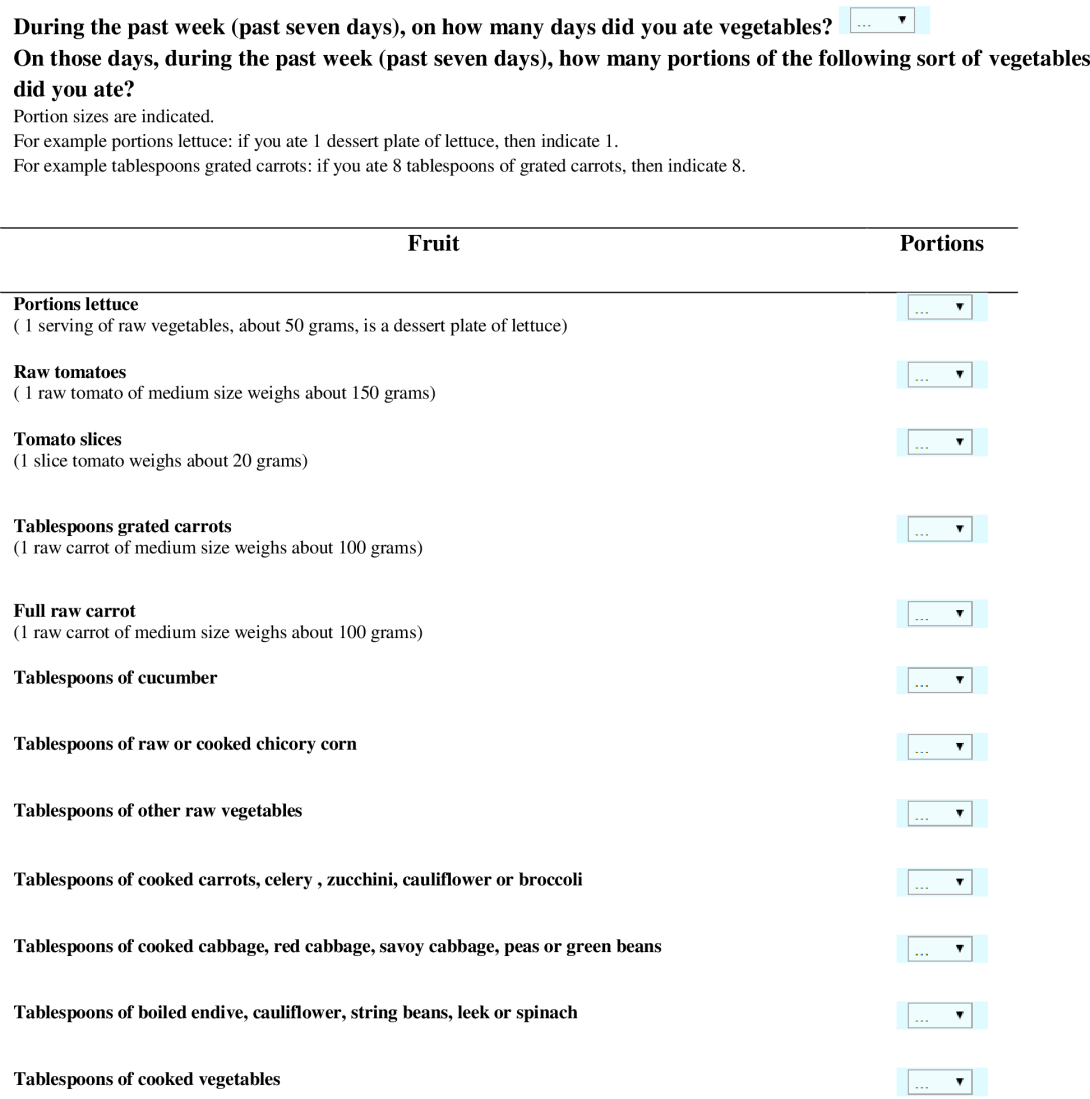
Vegetable Test. Items in the Vegetable Test are grouped based on similar portion sizes. Via the dropdown, participants can indicate the amount of portions of vegetables they ate.

Figs 1 and 2 illustrate the FT and VT, respectively. First, participants were asked on how many days in the past week (past seven days) they ate fruit (FT) and vegetables (VT). Next, if participants ate fruit/vegetables on one or more days, a list with frequently eaten fruits/vegetables in a Western diet was displayed on the screen. The displayed fruits and vegetables were based on the Belgian food table and consumption data[21]. For each type of fruit or vegetable, portion sizes and household sizes were mentioned (e.g. 1 cherry = 4 gram, 1 dessert plate of berries weighs about 100 grams). Participants were instructed to indicate for each type of fruit or vegetables the number of portions they ate during the past seven days.

The reported portions of fruit/vegetables were multiplied with the portion size of the corresponding types of fruit/vegetables to calculate the average grams per week. To calculate the average portion size per day, the average grams per week were divided by seven (see formulas in Table 1). Portion sizes and household sizes were based on prescriptions of the Belgian Superior Health council[23].

**Table 1:** formula total portion of vegetables and fruit per day.

**Calculation total portion of vegetables per day**	((portion_lettuce)*50 + (portion_raw_tomatoes)*150 + (portion_slices_tomatoes_1)*15 + (portion_raw_carottes)*100 + (portion_spoons_carottes*20) + (portion_cucumber*40) + (portion_corn* 15) + (portion_other_rawvegetables * 25) + (portion_broccoli* 30) + (portion_cabbage* 25) + (portion_leek*40) + (portion_boiledvegetables*30))/7
**Calculation total portion of fruit per day**	((portion_fruitsalad*150) + (portion_apples*125) + (portion_berries*100) + (portion_strawberries*100) + (portion_figs* 50) + (portion_plums *60) + (portion_cherries *50) + (portion_kiwi *75) + (portion_nectarine*90) + (portion_mango* 200) + (portion_melon* 180) + (portion_litchis*11) + (portion_grapes*125) +(portion_freshfruit *30) + (portion_cannedfruit *40) + (portion_fruitcompote *30))/7

### 7-day food diary

Participants were instructed to weigh fruit and vegetables (in grams) and to record this every day at home in a 7-day food diary. Two dietitians reviewed the diaries with participants for completeness and coded the diaries. To ensure construct validity, definitions of fruit and vegetables types included in the FT and the VT were similar to definitions used to code vegetables and fruits species in the 7-day food diary. These definitions were derived from the Flemish active food triangle[22, 28]. Items in the FT and VT were grouped together based on similar portion sizes. If it was impossible to weigh vegetables or fruit (e.g. at a restaurant), participants were allowed to use household sizes (e.g. a spoon, half of a plate, slices, …). In case household sizes were used, the same portion sizes as used in the VT and FT were used to calculate the amount of grams (See Table 1). Total FV intake per day was measured by summing all recorded grams and calculated grams (based on household sizes) and by dividing this sum by seven.

### Statistics

SPSS21 (SPSS Inc., Chicago, IL, USA) was used to perform statistical analyses. The outcome variables (total fruit intake per day and total vegetable intake per day) were first checked for normality by plotting a normal score plot.

Intra class correlations (ICC) of total FV intake measured at week one and week two were used to evaluate reliability. To interpret the ICC values, ratings by Landis and Koch[29]: 0.00–0.20 (poor), 0.21–0.40 (fair), 0.41–0.60 (moderate), 0.61–0.80 (substantial), 0.81–1.00 (excellent) were used. Participants (n = 2) who did not complete the FT/VT two times were not included in the analysis.

Because the distribution of the data was not normal, non-parametric spearman rank correlations coefficients (ρ) were used to evaluate validity of the questionnaires. Correlations of 0.30 to 0.40 were considered as good validity, correlations of 0.20 to 0.30 were considered as moderate validity and correlations lower than 0.20 were considered as not valid[30]. In addition, absolute agreement between FV intake measured by the FT/VT and FV intake measured by the 7-day food diary was calculated. As the data were not normally distributed, we used a non-parametric approach, as suggested by Bland-Altman [31, 32]. A non-parametric plot was interpreted, one for fruit intake, and one for vegetable intake. For the plot of FT, the difference between fruit intake measured by the FT and measured by the 7-day food diary was calculated and expressed as a percentage of difference (reported fruit by the FT as a percentage of 7-day food diary data). This percentage of difference was plotted on the y-axis against the average of fruit intake derived via the 7-day food diary on the x-axis. The 5th and 95th percentiles of the percentage-of-difference measures were calculated and subsequently inserted on the scatter plot, reflecting 90% limits of agreement (LOA). An identical procedure was followed for vegetable intake.

## Results

### Participant characteristics

In total, 58 adults (31% male, M age = 30y, ±12.09) participated in this study. Only two participants did not fill in the FT and VT for the second time. Fruit was consumed on a daily basis by 43.1% of the adults. Daily average intake of fruit was 166.0 (±94.80) grams. About half of the participants (53.4%) ate vegetables on a daily basis and adults ate on average 139.9 (±82.99) grams of vegetables per day.

### Test-retest reliability

The test-retest reliability study (n = 56) indicated that the FT showed excellent reliability (ICC = 0.81) for vegetable consumption. The VT also showed substantial reliability (ICC = 0.78) for vegetable consumption.

### Criterion (concurrent) validity

**Fig 3:**
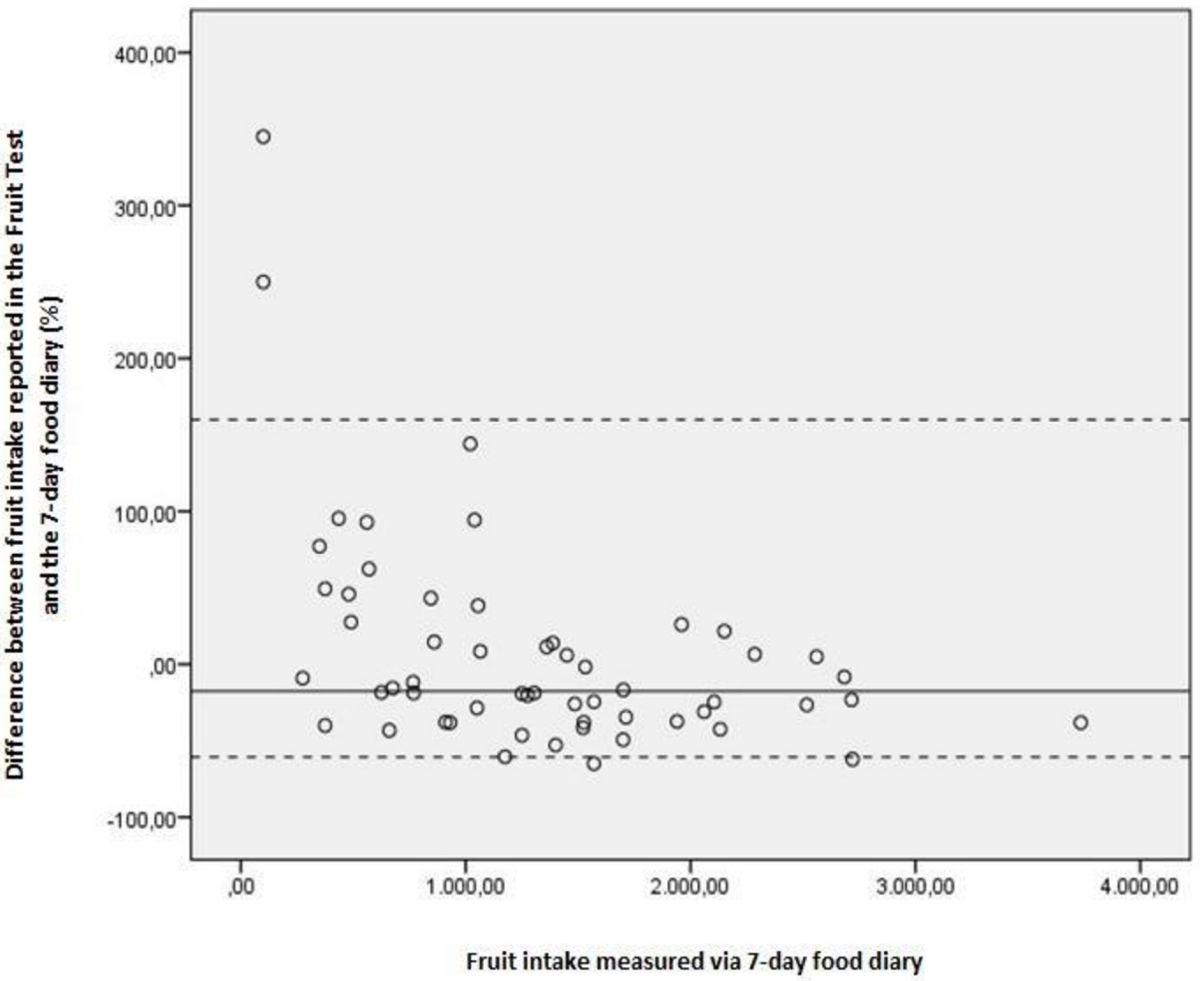
Non-parametric plot for fruit intake. Difference between fruit intake reported in the FT and the 7-day food diary. Y-axis represent these differences as a percentage of difference (reported fruit by the FT as a percentage of 7-day food diary data); x-axis represent fruit intake as measured by the 7-day food diary. Full lines represent median (M) percentage of difference; dotted lines show the 90% nonparametric limits of agreement (LOA), representing 5th and 95th percentiles (P5 and P95): M = -17,61; P5 = -60,68; P95 = 160,00).

Spearman rank correlation coefficients between self-reported fruit intake in the diary and self-reported fruit intake via the FT showed good validity (ρ = 0.73). In terms of percentage of difference between fruit intake reported in the FT and the 7-day food diary, the median difference was -17,61%. This indicates a small under-estimation of fruit intake in the FT against the 7-day food diary. Fig 3 provides the results on the criterion validity for the FT. Results indicate a modest variability in differences in fruit intake measured by the FT and the 7-day food diary. There is a higher variability at the lowest measured fruit intake in the 7-day food diary and lower variability at the highest measured fruit intake in the 7-day food diary.

**Fig 4:**
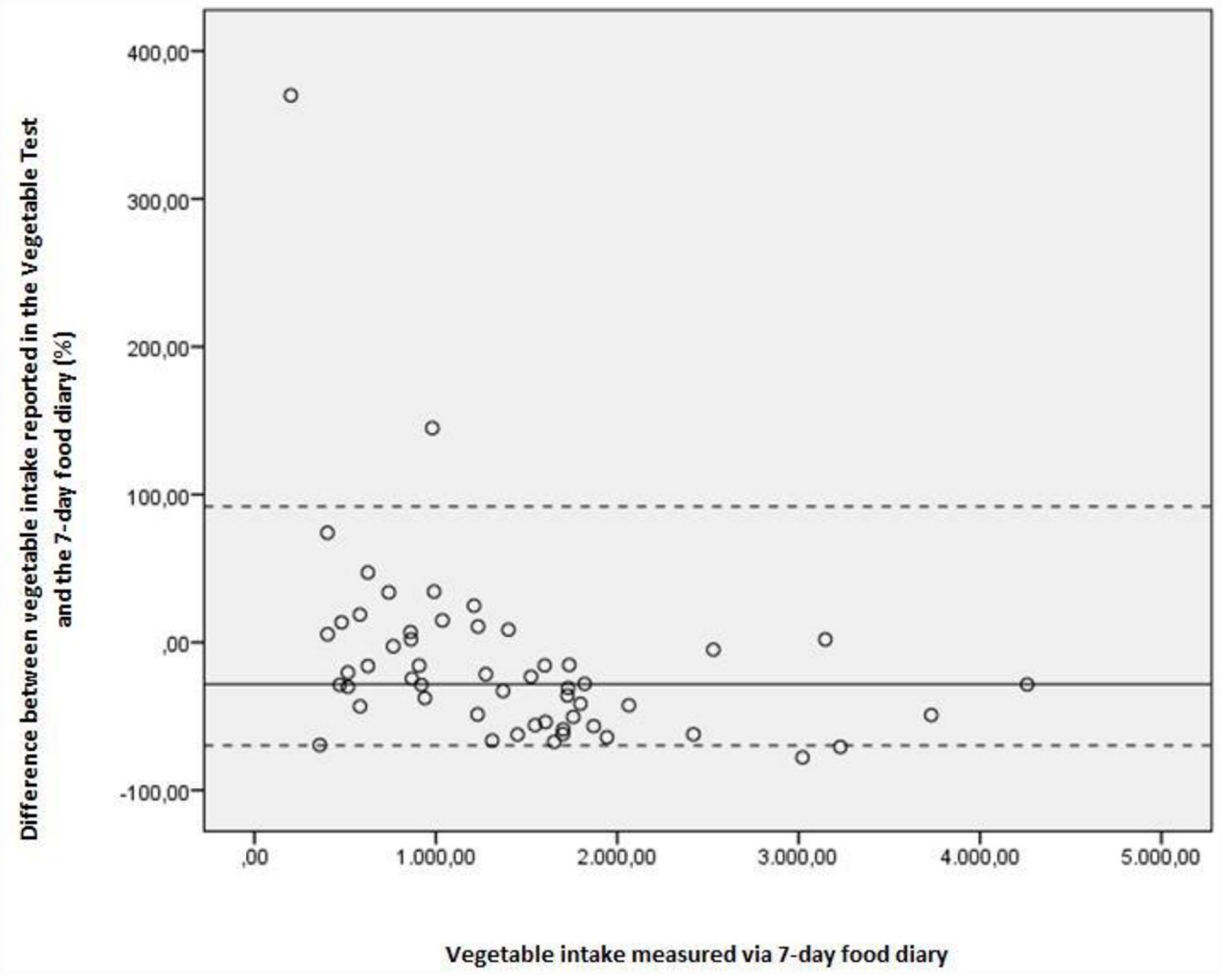
Non-parametric plot for vegetable intake. Difference in vegetable intake as measured by the VT and the 7-day food diary. Y-axis represent these differences as a percentage of difference (reported vegetables by the FT as a percentage of 7-day food diary data); x-axis represent vegetable intake as measured by the 7-day food diary. Full lines represent median (M) percentage of difference; dotted lines show the 90% nonparametric limits of agreement (LOA), representing 5th and 95th percentiles (P5 and P95): M = -28,27; P5 = -69,77; P95 = 91,82).

Comparison between self-reported vegetable intake in the diary and self-reported vegetable intake in the VT showed good validity (ρ = 0.52). In terms of percentage of difference between vegetable intake reported in the VT and the 7-day food diary, the median difference was -28,27%. This indicates a small under-estimation of vegetable intake in the VT against the 7-day food diary. Fig 4 illustrates the criterion validity results regarding the VT in more detail. It shows a modest variability in differences in vegetable intake measured by the VT and the 7-day food diary. There is a higher variability at the lowest measured vegetable intake in the 7-day food diary and lower variability at the highest measured vegetable intake in the 7-day food diary.

## Discussion

The short online FT and VT have already shown to be feasible questionnaires to measure FV intake in adults[20]. The aim of the current study was to test their test-retest reliability and criterion validity among Flemish adults.

Reproducibility of a questionnaire can be influenced by the questionnaire itself, respondents’ memory and actual changes in food intake[8]. Nevertheless, our results indicated that test-retest reliability of the FT and VT questionnaires was good.

Average fruit intake (166 gram/day) measured by the FT was comparable to average fruit intake in Europe (European Food Information Council (EUFIC, 2012) (166 gram/day)[33], but higher than in the Belgian Health Survey (average intake of 118 gram/day)[34]. Average vegetable intake measured with the VT (140 gram/day) was comparable to average vegetable intake in the Belgian health survey study (138 gram/day)[34], but lower than in the European Food Information Council (EUFIC) study in 2012 (220 gram/day)[33]. Good correlations ((FT (ρ = 0.73); VT (ρ = 0.52)) were found between FV intake consumption over seven days reported in the diet records and the FT and VT. A review on validated survey instruments of FV intakes in adults observed correlations around 0.40 to 0.50. However, these results should be cautiously interpreted, since they can only suggest that the FT and VT were relatively good at ranking individuals by their reported FV intake, when compared to FV intake reported in the 7-day food diary[35, 36]. Therefore, to evaluate validity of the VT and FT, Spearman rank order correlations were also combined with agreement plots. The plots for the VT and the FT showed a modest variability in differences in FV intake measured by the FT/VT and the 7-day food diary. These results also indicate a good validity of the FT and VT. Other validity studies of FFQs have reported that detailed food questionnaires containing many food items were not valid due to over-reporting of food consumption[5]. In contrast, the plots in our study showed a small under-reporting of FV intake measured by the FT and VT against the 7-day food diary. This may indicate that overestimation can be tempered by using brief questionnaires that include a moderate number of representative fruit and vegetable items, questions on portion size, mentioning household sizes with sufficient detail (e.g. 1 serving of raw vegetables = about 50 grams = a dessert plate of lettuce) and by including food items highly tailored to the dietary behaviors and local food items of the population surveyed[10]. Based on this study and the review of Kim and Holowaty (2003), we also suggest to further incorporate portion size pictures and consumption of mixed vegetables to decrease under-estimation in order to further improve validity of the FT and VT[10]. To use the FT and VT as measurement instruments in other countries, it might be that some adaptations are needed, since only commonly eaten fruits and vegetables in a Western diet (based on Belgian consumption and household purchase data) were included in the FT and VT[21–23]. The questionnaires can easily be adapted for the use beyond Europe (for example in an Asian diet), by adding other commonly eaten fruit or vegetables. However, to ensure generalizability, we recommend to re-evaluate reliability and validity of the questionnaires when adapting or translating it.

Some study limitations need to be acknowledged. In this study, both the FT and VT and the diet record (golden standard) were subjective methods to measure FV intake. This can lead to social desirability and recall bias [12, 37, 38]. To prevent social desirability and recall bias, it is often suggested to measure fruit and vegetable objectively, by using biomarkers(e.g. plasma concentrations of vitamin C)[8, 38]. However, biomarkers also have some limitations. Participants need to provide blood samples and plasma concentrations of carotenoids and vitamin C are also influenced by biologic factors, plasma cholesterol, body mass index, sex, smoking and vitamin supplement use, which can lead to modest validity[8, 38, 39].

Another limitation of our study is that we did not take into account certain individual factors that may have affected the results, such as socio-economic status. Since we used a convenience sample of adults, our sample may have consisted mainly of adults with a high socio-economic status. Adults with lower SES might experience more difficulties to fill in the FT and VT, which can influence validity of the questionnaires when they are used in these populations.

The FT/VT and the 7-day food diary were also not assessed at the same time, which makes it impossible to disentangle what is the variation in the two measures that is caused by the instrument itself and what is the variation that may have been caused by variations in diet in two different weeks [12, 40, 41]. But, if the diary is used before or at the same time when the FT and VT are assessed, the recording process in the diary could improve the recall of fruits and vegetables in the VT and FT. This may augment the correlations between results of the two methods[24, 42, 43]. To limit variation in diet in two different weeks due to other influences (e.g. changes in seasons, influences from advertisements), we kept the study period as short as possible. The study was conducted in the winter only and a short period (1 week) was used between the test-retest administrations.

In conclusion, this study shows that the FT and the VT are valid and reliable tools to measure FV intake in adults. Since these online questionnaires were already been evaluated as feasible (i.e. having a good length, easy to fill in and clear), they can be considered as valuable tools to measure FV intake of adults in future eHealth interventions. However, when using the questionnaire in different population groups, a re-evaluation of the reliability and validity of the questionnaire is recommended.
